# Large scale phosphoprotein profiling to explore *Drosophila* cold acclimation regulatory mechanisms

**DOI:** 10.1038/s41598-017-01974-z

**Published:** 2017-05-10

**Authors:** Hervé Colinet, Charles Pineau, Emmanuelle Com

**Affiliations:** 1 Université de Rennes 1, UMR CNRS 6553 ECOBIO, 263 avenue du Général-Leclerc, 35042 Rennes, France; 2 Protim, Inserm U1085, IRSET, Campus de Beaulieu, 35042 Rennes, France

## Abstract

The regulatory mechanisms involved in the acquisition of thermal tolerance are unknown in insects. Reversible phosphorylation is a widespread post-translational modification that can rapidly alter proteins function(s). Here, we conducted a large-scale comparative screening of phosphorylation networks in adult *Drosophila* flies that were cold-acclimated *versus* control. Using a modified SIMAC method followed by a multiple MS analysis strategy, we identified a large collection of phosphopeptides (about 1600) and phosphoproteins (about 500) in both groups, with good enrichment efficacy (80%). The saturation curves from the four biological replicates revealed that the phosphoproteome was rather well covered under our experimental conditions. Acclimation evoked a strong phosphoproteomic signal characterized by large sets of unique and differential phosphoproteins. These were involved in several major GO superclusters of which cytoskeleton organization, positive regulation of transport, cell cycle, and RNA processing were particularly enriched. Data suggest that phosphoproteomic changes in response to acclimation were mainly localized within cytoskeletal network, and particularly within microtubule associated complexes. This study opens up novel research avenues for exploring the complex regulatory networks that lead to acquired thermal tolerance.

## Introduction

Most ectothermic animals have the capacity to modify their thermotolerance to cope with environmental fluctuations. Pre-exposure to sub-lethal temperature triggers biochemical and physiological adjustments that usually promote subsequent thermal tolerance, a phenomenon referred to as thermal acclimation^1, 2^. Like many species, the fruit fly *Drosophila melanogaster* has the capacity to enhance thermotolerance in response to acclimation and this plastic response has been well described for both heat and cold^2, 3^. Several forms of acclimation exist (rapid, gradual, or developmental) that differ according to the timing and the length of the pre-exposure^1–4^. Recent data suggest that the genetic architecture of different forms of acclimation are non-overlapping, even though associated genes share some mechanistic similarities^4^. The physiological underpinnings responsible for cold acclimation are still under deep investigation, in particular, the regulatory mechanisms underlying acclimation are mostly uncharted territory^5^.

In insects, several studies have explored the underpinnings of thermal acclimation at transcriptional and translational levels^4–9^. Collectively, these data suggest that acclimation is tightly regulated at various biological levels, from gene expression to protein abundance. Despite the relevance of post-translational modifications (PTMs) for protein function, the degree to which the posttranslational regulatory network determines thermal acclimation has not yet been investigated in insects. Only recently, gel-based phosphoproteomic analysis revealed multiple changes related to rapid cold hardening (RCH) in the flesh fly^5^ which reinforces the notion that reversible phosphorylation is a major contributor to the phenotypical acquisition cold tolerance. In plants, PTMs are well known to be critical for regulating cold acclimation or freezing tolerance and different lines of evidence suggest that the disruption of phosphorylation deeply alters the ability of plants to acclimate to low temperature^10–12^.

Protein phosphorylation, a network of protein kinases and phosphatases and their respective protein substrates, is a pervasive regulatory mechanism that plays pivotal roles in controlling most of cellular processes^13^. The mechanism is ubiquitous throughout animals and plants, and countless proteins are phosphorylated by hundreds protein kinases^13^. For example, the Human Protein Reference Database lists over 95,000 phosphosites mapped to more than 13,000 proteins and approximately, 1–2% of human and eukaryotic genes encode protein kinases^14^. Many forms of adaptation in response to changing environmental conditions are regulated by phosphorylation events, and thus, changes in phosphorylation networks play key role in generating phenotypic plasticity^15^. Regulation by phosphorylation may be particularly important when rapid cellular changes are needed, as in the case of thermal acclimation, because transcription and translation are limited by the time needed for processing RNA molecules and proteins^15^. In insects, it has been reported that reversible phosphorylation plays pivotal role in mediating cold hardiness over winter season. However, these conclusions are based on targeted studies performed on kinases and phosphates^16, 17^. So far, shotgun phosphoproteomics of thermal acclimation has not yet been performed in insects. Previous 2D-DIGE proteomic studies have suggested that cold acclimation may involve posttranslational regulation in addition to *de novo* protein synthesis^8, 9^ and recent data based on Pro-Q Diamond 2D gel staining further support this view^5^. The availability of sensitive instrumentation and the development of new chromatography techniques to enrich phosphopeptides now allow shotgun phosphoproteomic analysis to be conducted, potentially allowing the detection of hundreds of phosphorylation events in a single experiment. These recent technologies could help in discovering the main targets of the reversible phosphorylation networks leading to thermal acclimation.

**Figure 1:**
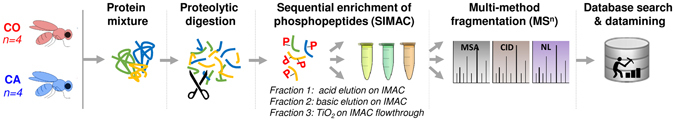
Schematic illustration of the **s**trategy used for comparative phosphoproteomic analysis of *D. melanogaster* adults that were cold acclimated (CA) versus control (CO). For each replicate (*n* = 4), lysate was collected from 20 virgin females and 4 mg of proteins were subjected to proteolytic digestion. Tryptic peptides were desalted and enriched for phosphopeptides using three sequential steps: IMAC with an acidic elution (fraction 1), IMAC with basic elution (fraction 2), and TiO_2_ on the IMAC flow through (fraction 3). Three different MS methods were then used for each elution fraction: a classical Collision-Induced Dissociation (CID), a Neutral Loss (NL) and a Multi-Stage Activation (MSA).

Here, we used an experimental strategy (illustrated in Fig. 1) adapted from SIMAC method^18^ to allow a large-scale identification of phosphopeptides and phosphoproteins from *Drosophila* samples during cold acclimation. Our goal was to conduct a hypothesis-generating shotgun phosphoproteomic approach as a pioneer exploration of the acclimation-induced changes in the phosphorylation networks.

## Results

### Cold acclimation promotes cold tolerance

**Figure 2:**
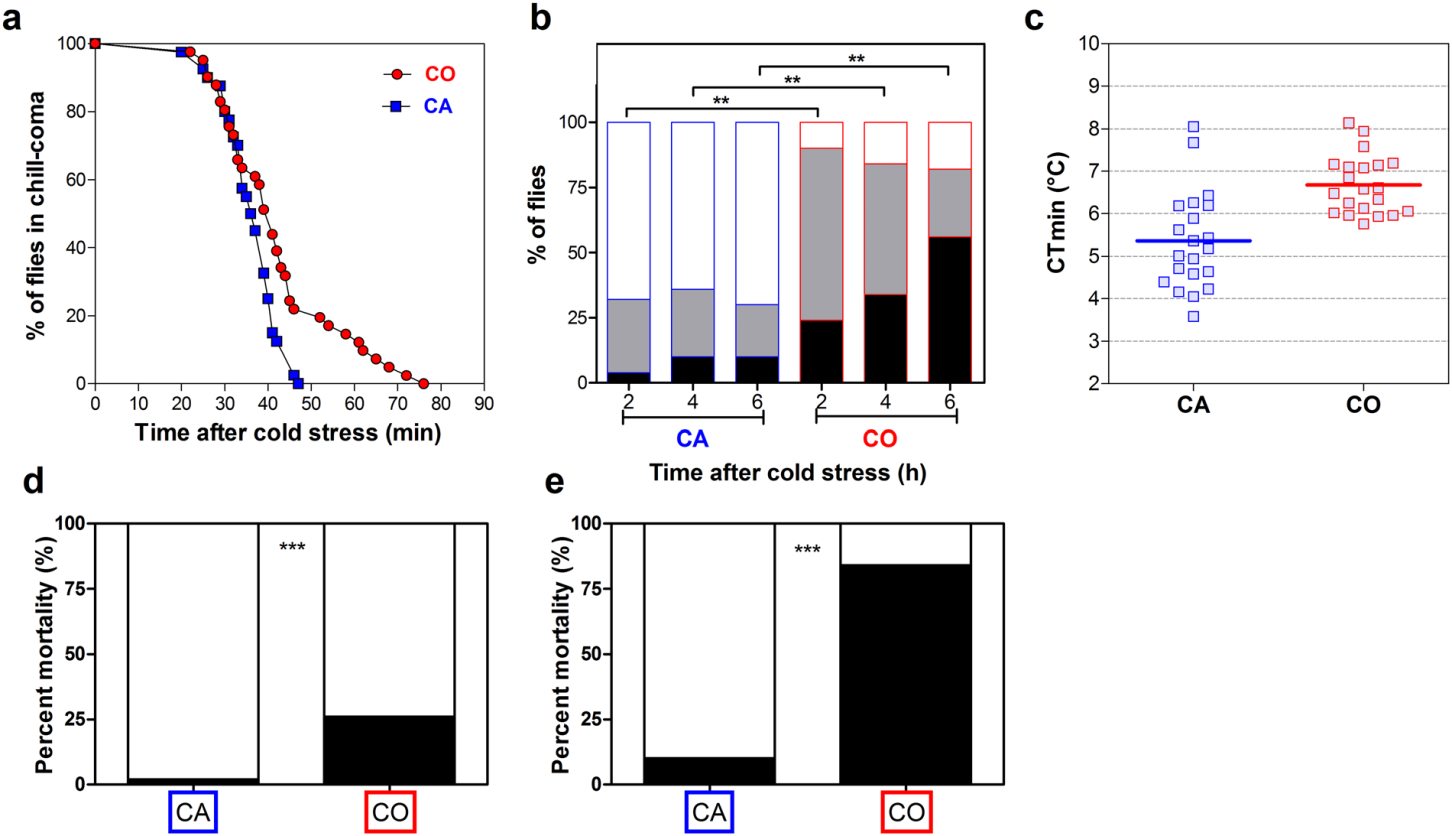
(**a**) Comparison of chill coma recovery dynamics in CA (blue squares) vs. CO (red circles) flies. Time to recover from chill coma was monitored in flies recovering at 25 °C after 9 h of chronic cold stress at 0 °C (*n* = 40). (**b**) Climbing activity monitored in CA vs. CO flies recovering flies after 2, 4 and 6 h following 9 h at 0 °C. Flies were categorized as fit (white part) or recovering (grey part) or injured (black part) (*n* = 50). (**c**) Critical thermal minimum (CT_min_) in the two groups tested. The horizontal lines indicate the mean value (*n* = 21). Mortality rate (black part) in CA and CO flies recovering for 24 h at 25 °C after 9 h of chronic cold stress at 0 °C (*n* = 50) (**d**) or 2 h of acute cold stress at −3.5 °C (*n* = 50) (**e**). Symbols (*) indicate a significant difference (*p* < 0.05).

First, we investigated how gradual cold acclimation (for five consecutive days) affected cold tolerance of adult flies. Different metrics were used to assess cold tolerance between cold acclimated (CA) and control (CO) flies. Chill coma recovery (CCR) patterns showed that CA flies recovered from cold stress much faster than CO flies (*Chi²* = 6.56, *p* = 0.01) (Fig. 2a). The climbing activity tests showed that CA flies regained activity faster than CO flies, and this was observed for all tested recovery durations: 2 h (*Chi²* = 36.39, *p* < 0.001), 4 h (*Chi²* = 24.73, *p* < 0.001) and 6 h (*Chi²* = 35.90, *p* < 0.001) (Fig. 2b). Mean CTmin varied between both groups (*t* = 4.49, *p* < 0.001) and was more than a degree lower in CA flies (5.3 ± 0.25 and 6.6 ± 0.15 °C for CA and CO, respectively) (Fig. 2c). Finally, post-stress survival was higher in CA than in CO flies after both chronic (98 vs. 74% survival in CA vs. CO; *Chi²* = 10.05, *p* = 0.002) and acute cold stress (90 vs. 16% survival in CA vs. CO; *Chi²* = 52.03, *p* < 0.001) (Fig. 2d,e). All metrics thus confirmed that cold acclimation deeply promoted cold tolerance, as previously reported^2–4^. We then tested whether this clear phenotypic change was associated with detectable changes in phosphorylation network.

### SIMAC allows the identification of a large number of phosphopeptides

**Figure 3:**
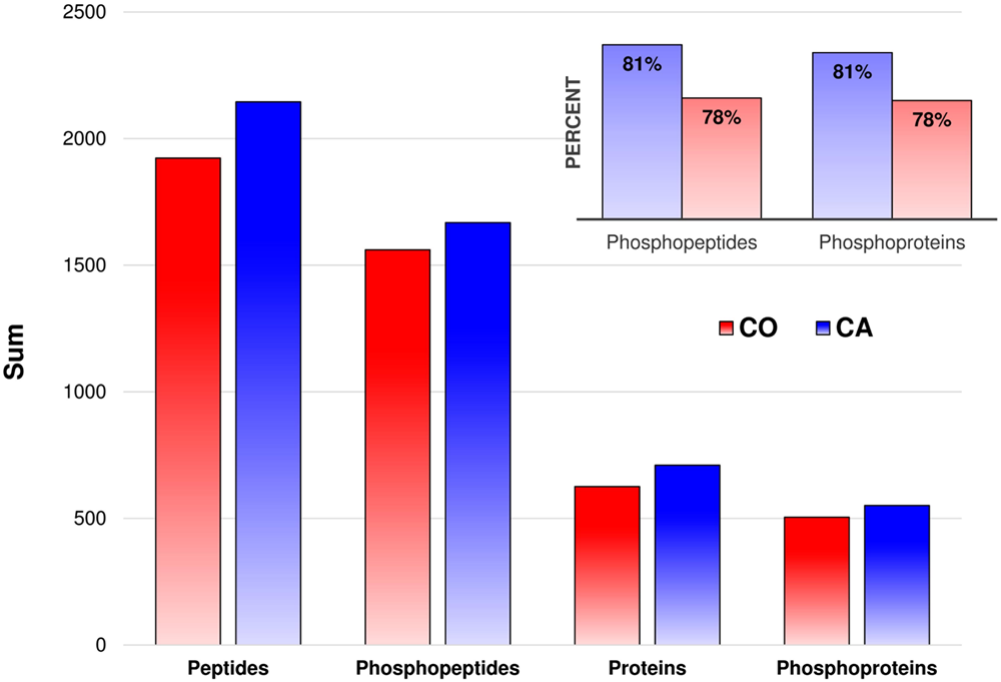
Bar plot summarizing phosphoproteomic yield and enrichment efficiency retrieved from the inference lists created at peptide and protein level for CA (blue) and CO (red) treatments. The bars show the total number of non-redundant identified peptides, phosphopeptides, and the corresponding number of identified proteins and phosphorylated proteins. The figure on top right shows the efficiency of enrichment corresponding to the ratio of phosphopeptides over peptides and phosphoproteins over proteins for CA (blue) and CO (red) treatments.

One of our goals in the present study was to adapt an appropriate workflow to detect and identify as many phosphopeptides (and phosphoproteins) as possible in fly’s samples. Our workflow (Fig. 1) consisted of four independent biological replicates per treatment (CA and CO). For each of these, tryptic peptides were enriched for phosphopeptides using three sequential steps: acidic elution on IMAC (fraction 1), basic elution on IMAC (fraction 2), and TiO2 on flow through (fraction 3). Each fraction was then subjected to three different MS methods to maximize the number of identified phosphorylated peptides: a classical collision-induced dissociation (CID), a Neutral Loss (NL), and a Multistage Activation (MSA) strategy. Using such workflow, we were able to detect 1923 and 2145 peptides in CO and CA flies respectively (from a protein inference list) (see Fig. 3). Of these, 81% and 78% were phosphorylated, which represented 1561 and 1668 phosphopeptides in CO and CA, respectively (Fig. 3). 97% were phosphoserine or phosphothreonine and 3% were phosphotyrosine in both treatments. This distribution of phosphorylated amino acids corresponds to patterns reported in previous phosphoproteomic studies on *Drosophila* cells (e.g. 97% on Ser/Thr and 3% on Tyr)^19, 20^ or other invertebrates (e.g. 83/12/5% on Ser/Thr/Tyr)^21^. Additionally, 1112 (71%) and 1261 (76%) were monophosphorylated, and 449 (29%) and 407 (24%) were multiphosphorylated in CO and CA, respectively. MS data were individually submitted to protein identification (peptide rank = 1; FDR < 1% at the peptide spectrum match level). Comprehensive information regarding the identification of all phosphopeptides, as well as the probability of the localization of the modification(s) and the localization of the phosphorylation in the modified peptides are provided for each replicate samples and treatment in Supplementary Table S-1. It resulted that peptides were assigned to 626 and 710 different protein sets, of which 505 and 551 were phosphorylated for CO and CA, respectively (Fig. 3). Hence, our rate of phosphopeptide enrichment at the protein level was 81 and 78% in CO and CA, respectively (see Fig. 3). Summarized data on the total number phosphopeptides and phosphoproteins, as well as enrichment efficiencies and phosphopeptides classes are provided for the four biological replicates separately in Supplementary Table S-2.

**Figure 4:**
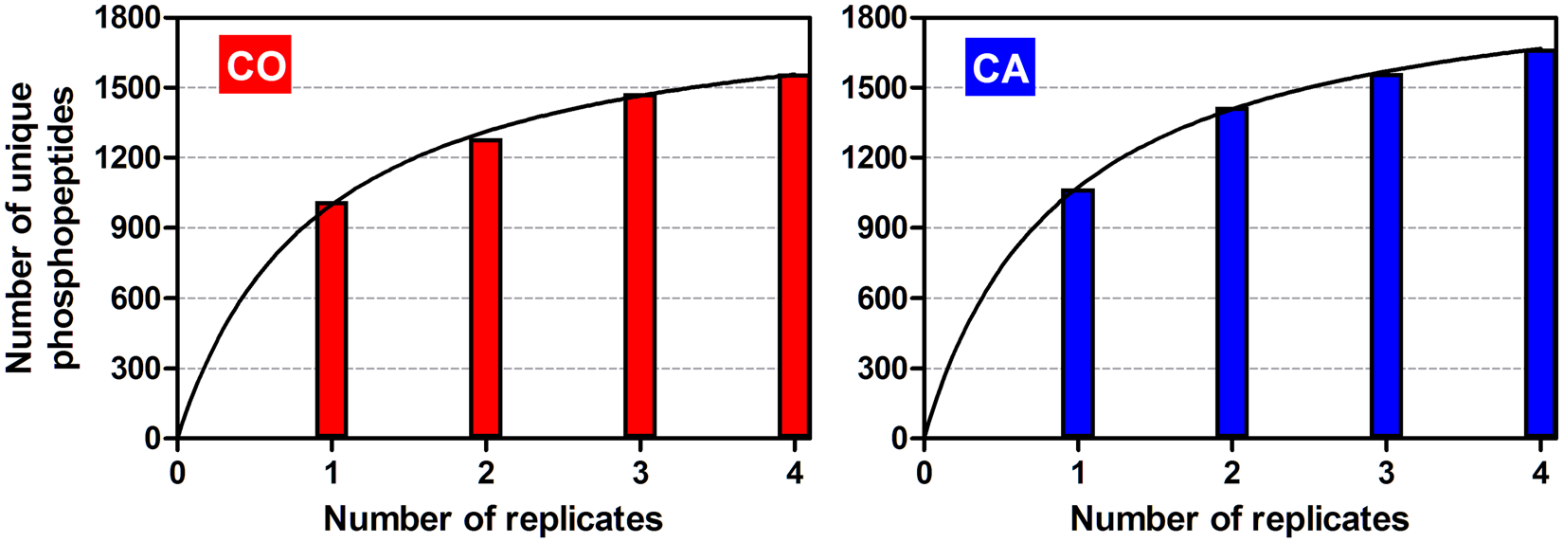
Saturation curves in CA (blue) and CO (red) conditions. The horizontal axis depicts the number of biological replicates and the vertical axis the cumulated number of unique phosphopeptides in each of these replicates. The lines represent non-linear curve fitting (details about the equation are in the text).

We investigated the reproducibility and coverage of our approach using saturation curves as described in Boekhorst *et al*.^22^. Briefly, for both treatments (CA and CO), we plotted the number of unique phosphopeptides identified against the number of replicates (4). In both treatments, we observed a plateau-shaped saturation curve (Fig. 4). We used the following non-liner equation to describe and fit the data (GraphPad Software, Inc., San Diego, CA, USA): Y = a*X/(*b* + X), where Y is the number of unique phosphopeptides, X the number of replicates, *a* is the estimated maximum and *b* correspond to a value to achieve a half-maximum. For both conditions, the *R²* was equal to 0.99 and the estimated value for *b* was inferior to 1 (0.90 and 0.91 for CA and CO, respectively), meaning that a single replicate was already enough to cover more that 50% of the total number of detected phosphopeptides. The replicate 2, 3 and 4 allowed respectively the detection of 32, 10 and 7% of phosphopeptides not yet identified in the earlier run in CA, and 26, 15, 5% in CO (Fig. 4). Hence, the curves combining the four replicates showed a stabilisation phase close to saturation, reflecting that the phosphoproteome was rather well represented under the specific conditions tested here.

### Phosphoproteomic changes in response to acclimation

**Figure 5:**
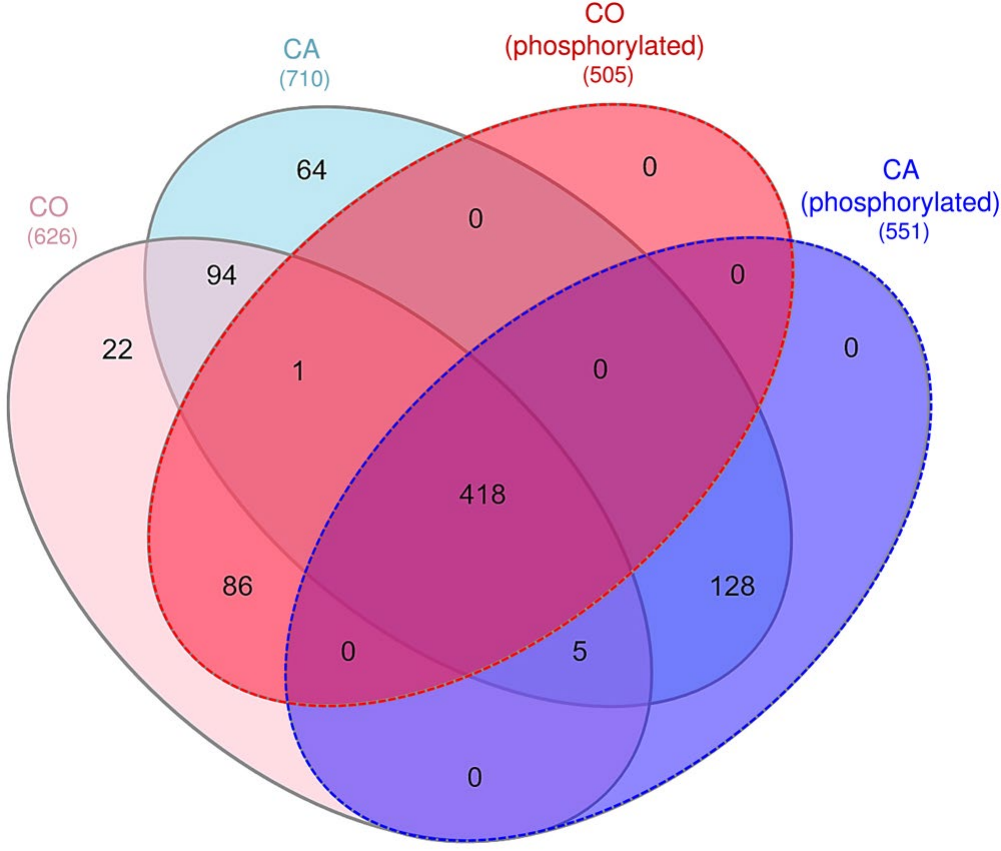
Venn diagram showing the distribution of proteins and phosphorylated proteins in CA (blue) and CO (red) conditions. Numbers in brackets indicate the number of entries in each list.

Two approaches were used to depict phosphoproteomic changes in response to acclimation. First, we identified sets of phosphorylated proteins uniquely expressed in each treatment and second, we detected phosphoproteins differently modulated in response to acclimation. For the first approach, were compared different lists of proteins: unphosphorylated and phosphorylated proteins identified in CO flies (626 and 505 IDs) and unphosphorylated and phosphorylated proteins in CA flies (710 and 551 IDs) (see Fig. 3). The overlaps among these sets are illustrated in the Venn diagram (Fig. 5). From these comparisons, we identified a set of phosphorylated proteins uniquely detected in CA flies (i.e., 133 phosphoproteins) and another set that was only present in CO and thus lacking in CA flies (i.e., 87 phosphoproteins). 418 phosphoproteins were detected in both CA and CO conditions. Unphosphorylated proteins were not considered in further analyses (22, 64 and 94 IDs in CA, CO and both, see Fig. 5).

**Figure 6:**
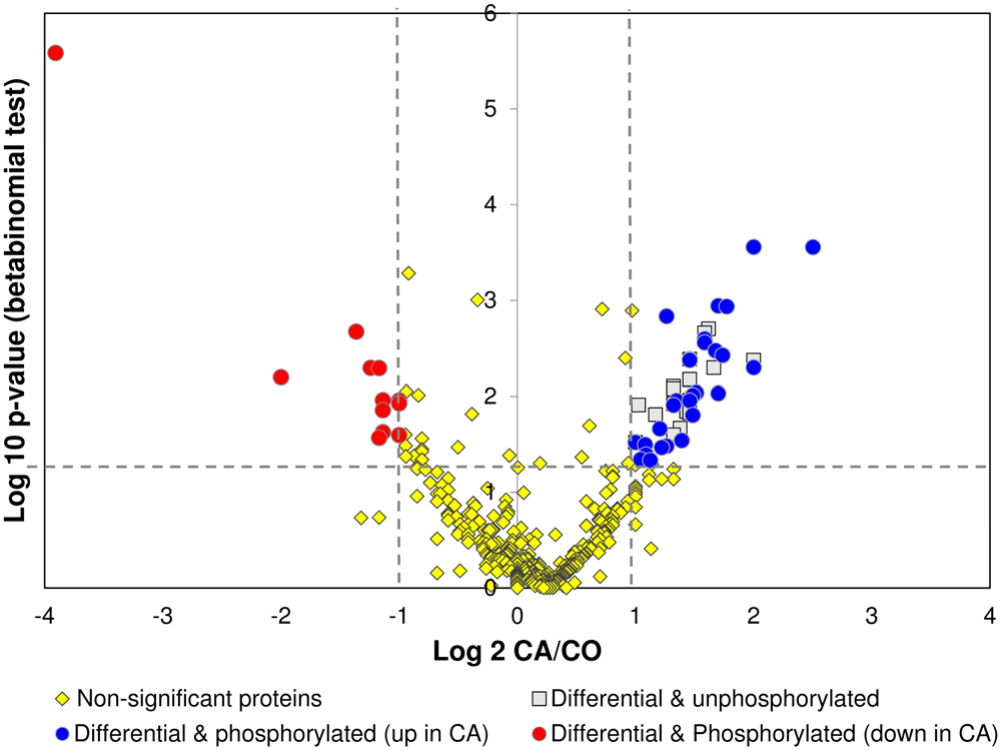
Volcano plot constructed from weighed spectral counts. The plot shows how much and how significant the identified proteins were differentially abundant between CA and CO flies. Horizontal dotted line depicts a *p* < 0.05 cutoff and vertical dotted lines depict 2- and 0.5-fold cutoffs. Among 64 significant differential proteins (i.e. above cutoff values), 18 were identified without phosphorylated peptides (grey squares), 12 were phosphorylated and less abundant in the CA samples (red circles) and 34 were phosphorylated and more abundant in the CA samples (blue circles).

For the second approach, weighted spectral counts were calculated in each biological sample for all proteins identified, which represented 811 proteins among which 633 were phosphorylated. We calculated an expression ratio (CA/CO) and a beta-binomial *p*-values, and these values were used to construct a volcano-plot which highlighted 64 significant differential proteins (with ratio > 2 and *p* < 0.05) (Fig. 6). Among these differential proteins, 46 were identified with phosphorylated peptides, of which 12 were down regulated and 34 were upregulated in the CA samples (Fig. 6). A summary of these differential proteins in provided in Supplementary Table S-3a (with annotation, names and functions), and the full details of the quantitative information of each protein is also provided in Supplementary Table S-3b.

### Functional annotation

**Figure 7:**
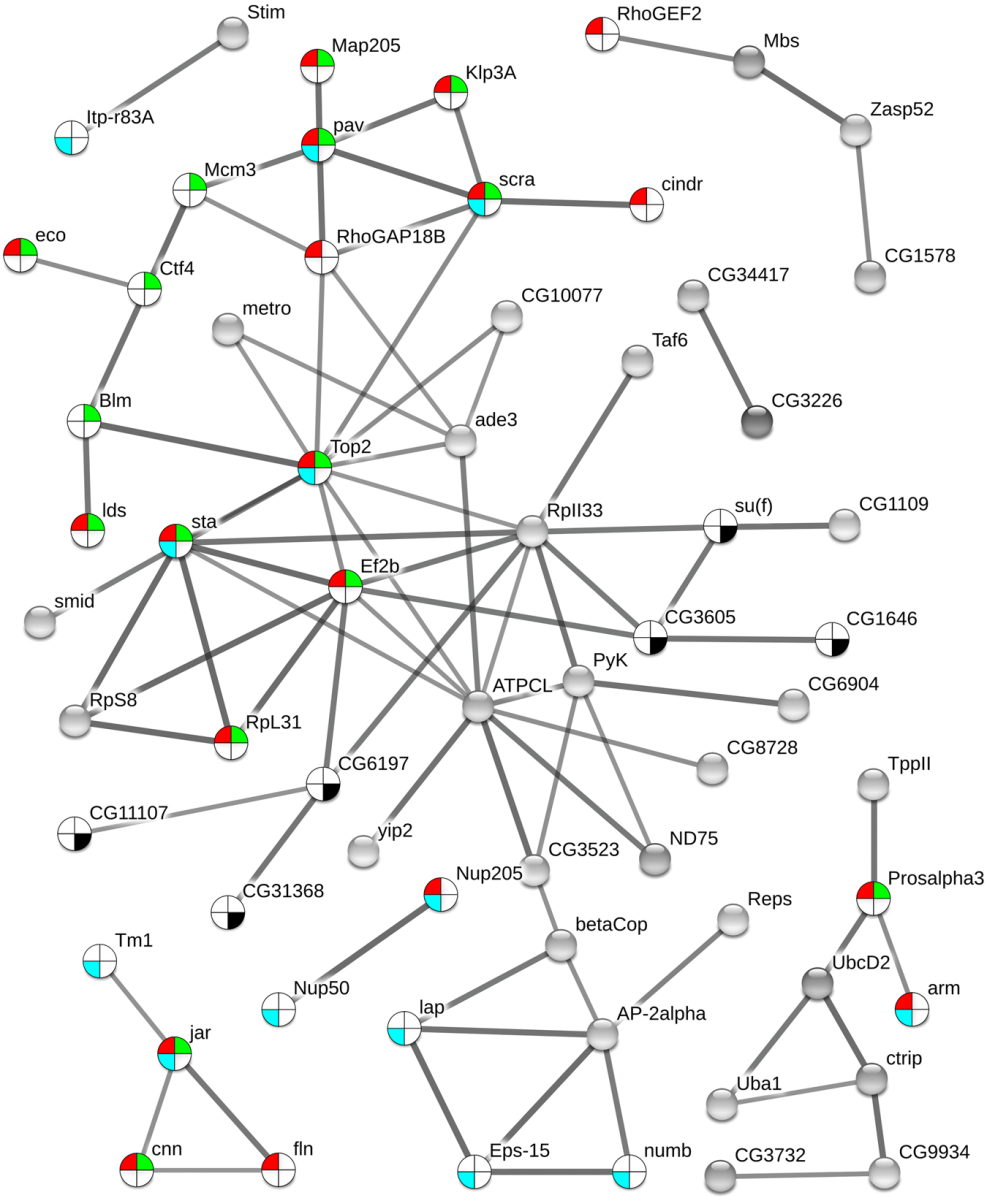
Phosphoprotein interaction network resulting from the set of proteins positively regulated by phosphorylation events [list CA^(+)^] with acclimation. The phosphoproteins set was analyzed for putative protein-protein interactions using STRING program with default settings except that we only considered high confidence interactions (with score > 0.7). Disconnected nodes are not shown in the network. Phosphoproteins involved in the four major GO-terms superclusters detected in REVIGO (see Fig. 8) are highlighted within the network with different colors: cytoskeleton organization (red), mitotic cell cycle (green), cellular localization (blue), and mRNA processing (dark).

**Figure 8:**
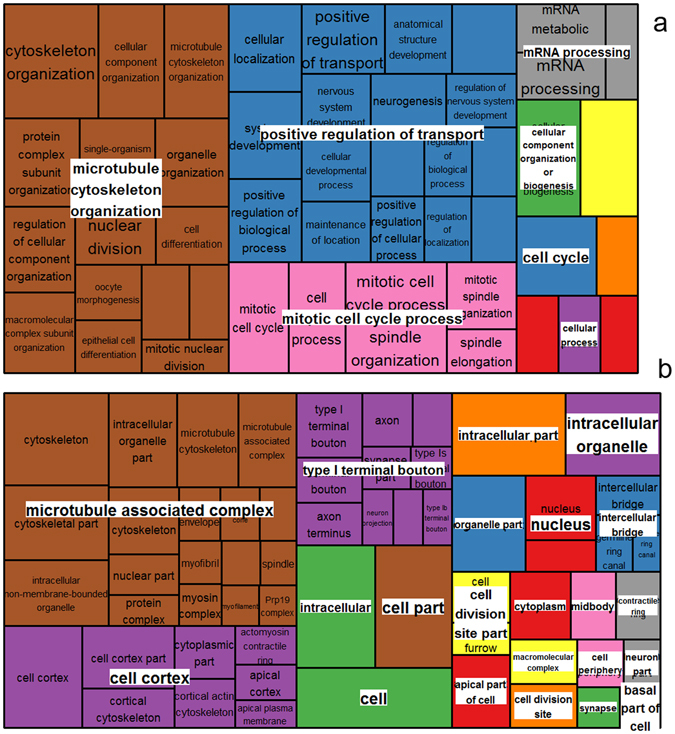
Illustration of superclusters of overrepresented GO-terms visualized in semantic similarity-based treemap views from REVIGO program, for biological process (**a**) and cellular component (**b**). Rectangles in the treemaps are size-adjusted to reflect the corrected *p*-value (i.e. larger rectangles represent the most significant GO-terms). Each rectangle in the treemap view has a single cluster for representation. These representatives are further joined together to build superclusters that are related terms and displayed in different colours.

Two different phosphoprotein sets were used for functional annotation: 1) list_CA^(+)^ comprised phosphoproteins uniquely detected in CA flies (133; see Fig. 5) and the differential phosphoproteins upregulated in CA (34; see Fig. 6). This list thus represents proteins that were positively regulated by phosphorylation events in response to cold acclimation. 2) list_CA^(−)^ comprised phosphoproteins absent in CA (87; see Fig. 5) and phosphoproteins downregulated in CA (12; see Fig. 6). Hence, this list represents proteins that were regulated by dephosphorylation events in response to cold acclimation. The list_CA^(+)^ and list_CA^(−)^ are provided in Supplementary Table S-4 together with annotation, names, symbols and function(s). These two protein sets were used to query STRING database^23^ to reveal possible protein-protein interactions. As shown in Fig. 7, the phosphoproteins from the list_CA^(+)^ had significant associations and intricate interactions (PPI enrichment *p*-value = 7 e^−06^) which indicates that, in response to acclimation, many phosphorylation events occurred on proteins that were clearly functionally related. Gene Ontology (GO) enrichment analyses performed on list_CA^(+)^ in STRING (considering FDR < 0.05) resulted in many overrepresented GO-terms: 48 biological processes, 7 molecular functions, and 61 cellular components. The full list of enriched GO-terms is provided in Supplementary Table S-5. To facilitate interpretation, the long lists of significant GO-terms was imported in REVIGO program^24^ to reduce the functional redundancy and depict presence of GO superclusters (based on semantic similarity). The hierarchical treemaps obtained from REVIGO program are shown separately for biological process (Fig. 8a) and cellular component (Fig. 8b). For the biological process, four major GO superclusters were detected: microtubule cytoskeleton organization (comprising the most significant GO-terms, see Supplementary Table S-5), positive regulation of transport, mitotic cell cycle and mRNA processing. Within the STRING network, it can be seen that the subsets of phosphoproteins specifically involved in these four GO superclusters were functionally connected (Fig. 7). This suggests coordinated phosphorylation events regulating these likely important biological processes for cold tolerance acquisition. Reduction of the cellular component GO-terms in REVIGO (Fig. 8b) revealed that phosphorylation events occurring in response to acclimation were mainly localized within the microtubule associated complex and within the cell cortex which primarily contains actins network. This supports that multiple phosphorylation-dependent regulations occurred within cytoskeletal structures in response to acclimation. The enriched GO terms for molecular function further supported this view with actin, microtubule and cytoskeletal protein binding being strongly over-represented (Supplementary Table S-5).

Analyses on the phosphoproteins from list_CA^(−)^ revealed no significant functional interactions in STRING (PPI enrichment *p*-value = 0.104), and only a few vague GO-terms were highlighted (cellular component biogenesis and cellular component assembly) (Supplementary Table S-5). Hence, it appears that sets of proteins dephosphorylated in response to acclimation did not target some specific biological functions or sets of functionally connected proteins.

## Discussion

In the present study, we investigated how cold acclimation affected cold tolerance of *D. melanogaster*. All metrics confirmed that acclimation deeply promoted cold tolerance. We hypothesized that this marked phenotypic change would be associated with altered posttranslational regulation. We conducted a first large-scale shotgun phosphoproteomic analysis to explore which processes and functions were regulated by phosphorylation events in response to cold acclimation in fruit flies.

We detected many cytoskeleton-related phosphoproteins that were unique in CA flies. Some phosphoproteins like microtubule-associated protein 205 and 60 (Map205 and Map60), transcription termination factor 2 (Lds), stathmin (Stai), tropomyosin-1 (Tm1) and flightin (Fln), had phosphorylated forms that were clearly more abundant in CA flies (Tables S-3a). Fln plays roles in contractile activity by modulating actin-myosin interaction^25^. Tm1 is also a muscle-related protein. We previously found changes in the abundance of different isoelectric variants of Fln in response to thermal acclimation^9^, and because Fln is regulated by phosphorylation^26^, we suspected acclimation-related phospho-regulation resulting in different phosphovariants. Here, we confirm that Fln is subjected to marked phosphorylation changes in response to acclimation. Our data also supports recent genomic study which suggested that regulation of cytoskeleton is important component of cold acclimation in a variety of tissues beyond muscle contractile apparatus^6^. We detected many different microtubule-associated proteins; some were again more phosphorylated in response to acclimation (Map60, Map205 and Stai), suggesting a phospho-regulation of microtubule cytoskeleton organization. Functional annotations revealed that cytoskeletal proteins or proteins involved in their assembly/disassembly were particularly targeted by phosphorylation regulation in response to adult cold acclimation. Interestingly, this is in very good agreement with data recently published by Teets and Denlinger^5^ who found differentially phosphorylated proteins involved in cytoskeleton organization as major driver of RCH in flesh fly. Despite marked differences between RCH (i.e. short term response) and adult acclimation (gradual response)^1–3^, some mechanistic overlaps occur between these two phenotypic responses to temperature^4^, and our data suggest that phosphorylation-mediated cytoskeleton reorganization represents thus a shared and conserve mechanism of cold tolerance acquisition and plasticity. Phosphorylation is a prerequisite for rapid modulation of cytoskeleton^27^ and studies on plant cold acclimation have also emphasized the importance of cytoskeleton remodelling in conferring tolerance to low temperature. Kerr and Carter^28^ reported that low temperature causes microtubule depolymerisation in winter rye root tips and that the level of depolymerisation is related to the degree of cold tolerance. Cold acclimation also triggers rapid up-regulation of actin-binding proteins in plants and other organisms, which indicates that the reorganization of the intracellular cytoskeleton structure is required for cold tolerance acquisition^29, 30^. In insects, a few targeted studies have addressed the importance of cytoskeleton reorganization in cold tolerance. The assembly/disassembly of microtubules and actin filaments is involved in diapause and cold response of *Culex pipiens* ^31, 32^. In *Delia antiqua*, actin depolymerisation occurs in chill-susceptible pupae, whereas this effect is mitigated in cold acclimated counterparts^33^. Cold treatment of certain cultured *Drosophila* cells induces reversible disassembly of microtubule arrays^34^. Insect proteomic and genomic data also detected changes in cytoskeleton genes or proteins correlated with cold tolerance^4, 6, 35^. A cytoskeleton remodelling is presumably fundamental to maintain the structure, function, and organization of cells upon low temperature. Our observation together with recent phosphoproteomic report^5^ strongly support that phospho-regulation plays a crucial role in cold-induced cytoskeleton remodelling.

Many unique or differential phosphoproteins involved in cellular localization and transport were detected in CA flies. This was the case for AP-2alpha which is involved in protein transport and endocytosis, and Tud which is involved intracellular mRNA localization. These two proteins were amongst the most differential phosphoproteins in CA flies (fold changes > 3). The functional annotation analysis underscored a supercluster related to positive regulation of transport, suggesting that phosphorylation events occurred on many proteins related to this process. Similarly, Teets and Denlinger^5^ also found several GO-terms related to cellular transport. This observation suggests a phospho-regulation on the trafficking of substances (e.g. macromolecules, small molecules, or ions) within or between cells in response to cold acclimation. To a great extent, the subcellular localization of proteins dictates their function. In consequence, phosphorylation-dependent subcellular trafficking is a way protein function(s) can be regulated^11^. Processes related to cellular transport were also altered in flesh fly submitted to cold stress and rapid acclimation^36^. Previous transcriptomic data in plants also detected an overrepresentation of transcripts involved in cellular transport and trafficking in response to cold acclimation^37, 38^. Regulated protein localization is also a fundamental principle of signalling. Long range movement of activated signalling proteins within the cell is regulated by phosphorylation that triggers the translocation of proteins in and out of the nucleus^13^. Variations in the phosphorylation level of proteins involved in cellular localization and transport could thus be related to the regulation of proteins function and/or signalling in response to acclimation; however, the specific targets of this cellular trafficking remain to be investigated. Both intracellular trafficking and signalling are intimately linked to cytoskeleton because both rely on diffusion along cytoskeletal tracks^39, 40^. Consequently, coordinated phospho-regulation on proteins related to cytoskeleton reorganization and cellular localization is likely required for cellular trafficking and signalling in response to acclimation.

RNA processing was also highlighted as supercluster in our functional analyses. During cold acclimation, transcripts need to be processed, exported, kept in a functional conformation and then degraded. RNA can fold into extensive secondary structures that could interfere with its function, and this interference can be exacerbated under low temperature^41^. We found several phosphoproteins, such as lariat debranching enzyme (ldbr), uncharacterized protein isoform D (CG31368) and GH10652p (CG10077), that were involved in RNA processing or splicing, and all exhibited phosphorylated forms more abundant in CA flies. Of particular interest, GH10652p was the most significantly regulated phosphoprotein in our quantitative analysis with more than 5-fold increase in CA flies. Interestingly, this protein is DEAD box RNA helicase. In plants and bacteria DEAD box RNA helicases are upregulated by cold acclimation and they function as RNA chaperones^41–43^. In bacteria, DEAD box RNA helicases are essential for stabilization and decay of mRNAs under low temperature^44, 45^. Phosphorylation of RNA helicase is a common physiological response to abiotic stress in plants that regulates its expression and activity^45^. Several analyses in plants^41, 43^, bacteria^44, 45^, and fishes^46^ have revealed the involvement of RNA processing and export in cold signalling and cold tolerance, and our data suggest that phospho-regulation on proteins related to RNA processing and nucleocytoplasmic transport may also play a role in insect responses to cold acclimation. Further studies should be conducted to depict the role of RNA helicase and its regulation in insect’s response to temperature change.

Many proteins regulated by phosphorylation were also involved in cell cycle. Minichromosome maintenance 3 protein (Mcm3), Map205 and elongation factor (EF2) exhibited phosphorylated forms significantly more abundant in CA flies. Cold acclimation is mediated by alteration in the mRNA and proteins present in cells, and these alterations directly affect cell proliferation and cell cycle progression^47^. EF2 was centrally located in the STRING network, suggesting it may play a central role in acclimation signalling and not only in cell cycle regulation. The abundance of this protein was previously shown to respond to thermal acclimation^8^. At mild low temperature, the translation is supposed to be reduced^47^ and this occurs via the well characterized phosphorylation of EF2^48, 49^. Reduced activity of translational machinery is a typical response to low temperature in prokaryotes and eukaryotes and is a direct consequence of altered cytoskeleton organization^48^.

## Conclusion

Reversible phosphorylation is one of the most crucial and widespread post-translational modifications of protein function. Using a modified SIMAC method followed by a multiple MS analysis strategy, we could identify a large collection of phosphopeptides and phosphoproteins. This workflow allowed high-throughput screening of dynamic changes in phosphorylation networks according to cold acclimation in *Drosophila* adults. To our knowledge, this study represents the first shotgun phosphoproteomic survey of adult fruit flies submitted to thermal treatment. It is noteworthy that cold acclimation evoked a strong phosphoproteomic signal, while protein abundance is hardly affected by the same treatments^8^. Our work suggests that acquired cold tolerance primarily involves coordinated series of phosphorylation events involving regulation of microtubule cytoskeleton organization, positive regulation of transport, mitotic cell cycle and mRNA processing. Although the precise regulatory mechanisms of acclimation are not yet known, these new data will be useful to pave the way for further targeted studies. Numerous phosphoproteins remain with functions that are either undefined or difficult to assign to a particular biological process. This emphasizes that the regulation which leads to complex phenotypes, including cold tolerance acquisition, remain far beyond our current understanding, even in model organisms such as the fruit fly.

## Material and Methods

### Fly culture and acclimation treatment

Flies from a laboratory population of *D. melanogaster* were used for this experiment. The population was founded from a large number individuals collected in October 2010 in Brittany, France. Flies were maintained in laboratory in 100 mL bottles at 25 ± 1 °C (light/dark: 12/12 h) on standard fly medium consisting of brewer yeast (80 g/L), sucrose (50 g/L), agar (15 g/L) and Nipagin® (8 mL/L). To generate flies for the experiments, groups of 15 mated females were allowed to lay eggs in 100 mL rearing bottles during a restricted period of 6 h under laboratory conditions. This controlled procedure allowed larvae to develop under uncrowded conditions. At emergence, flies were sexed to keep only virgin females. Sexing was done visually (with an aspirator) without CO_2_ to avoid stress due to anaesthesia^50^. Experimental females were left to age on food for 6 days (food was changed every two days) under standard conditions before they were assigned to the treatments. Females were synchronized at the age of 6-d-old to avoid confounding effects of young maturating adults (<3 day-old)^51^. These females were randomly exposed to either thermoperiodic cold acclimation or control conditions for five consecutive days. The abbreviations ‘cold-acclimated’ (CA) and ‘control’ (CO) are used to distinguish the two experimental conditions. The temperature fluctuated from 13 to 17 °C or from 23 to 27 °C (using light/dark 12 h/12 h cycles) for CA or CO respectively. Programmed thermo-regulated incubators (Model MIR-153, SANYO Electric Co. Ltd, Munich, Germany) were used and temperature was checked using automatic recorders (Hobo data logger, model U12–012, accuracy ± 0.35 °C, Onset Computer Corporation, Bourne, MA, USA). A 12 h/12 h photoperiod was used with the scotophase occurring during the cold period. A similar cold acclimation treatment has previously been used and successfully promoted cold tolerance^3^. After 5 days of thermal conditioning, adult flies were tested for cold tolerance at the end of a thermoperiod cycle (i.e. when temperature was at a minimum). Some females from both phenotypic groups were also snap-frozen in N_2_ and stored at −80 °C for phosphoprotein profiling.

### Cold tolerance assessment

Different metrics were used to assess the phenotypic changes (i.e. increased cold tolerance) resulting from cold acclimation. First, chill coma recovery (CCR) following nonlethal chronic cold stress was measured as previously described^3^. Briefly, 40 females were exposed to 0 °C for 9 h by placing a vial in a cold incubator (Model MIR-153, SANYO Electric Co. Ltd, Munich, Germany). Flies were then allowed to recover at 25 ± 1 °C, and recovery times were individually recorded. Flies were considered recovered when they stood up. Data were used to generate temporal CCR curves, which were compared between CO and CA with Mantel-Cox analysis. Second, climbing activity tests were used to assess the medium-term recovery as previously described^52^. Briefly, for each treatment (CA and CO), 50 flies were individually transferred to a 9.5 cm plastic vial and the height flies reached within 7 s after a mechanical stimulation was noted. Flies were divided into three categories: injured, recovering, and fit. This test was performed repeatedly on the same individuals after 2, 4 and 6 h of recovery (at 25 °C). Flies were maintained on food during this period. Chi square contingency tests were carried out to compare numbers of flies in the three categories. Survival of flies was also measured following (i) a chronic cold stress (0 °C for 9 h) and (ii) an acute cold stress (−3.5 °C for 2 h). In both tests, 5 replicated pools of 10 females (*i.e*., a total of 50 flies) were placed in 42 mL glass vials immersed in a circulating bath of ethylene glycol (Haake F3 Electron, Karlsruhe, Germany) set at the required temperature. After the stress, the flies from both phenotypic groups were returned to 25 °C on standard diet and the mortality was scored after 24 h. Chi square contingency tests were used to compare mortality rates between both groups. Finally, critical thermal minimum (CTmin) was investigated. An ethylene glycol jacketed glass cylinder (35 × 5 cm) was used. Temperature in the cylinder was controlled by circulating ethylene glycol from a programmable bath (Haake F3 Electron, Karlsruhe, Germany). Flies were cooled from 20 °C to the CTmin at 0.5 °C min^−1^. Upon entering chill coma, flies fell out and the temperature inside the column was recorded using thermocouple (type K, accuracy of ±0.10 °C). For each group, 21 females were tested for CTmin. Mean CTmin values were compared between CA and CO using *t*-test. Experimental groups of flies were different for all the different cold tolerance assays.

### Phosphoproteomics

#### Protein extraction

Protein extraction was performed as previously described with minor modifications^8^. Briefly, four biological replicates, each consisting of a pool of 20 virgin females, were used for both phenotypes (CO vs. CA). After grinding to fine powder in liquid nitrogen and precipitation with 10% trichloroacetic acid in acetone for 2 h at −20 °C, samples were lysed in 30 mM Tris buffer pH 7.4 containing 8 M urea, 4% CHAPS, protease inhibitors (Protease Inhibitor Mix, GE Healthcare, Vélizy Villacoublay, France), and phosphatase inhibitors (Halt^TM^ Phosphatase Inhibitor Cocktail, ThermoFisher Scientific, Illkirch, France), using an ultrasonic processor (Bioblock Scientific, Illkirch, France) as previously described^8^. After centrifugation (16,000 g for 20 min at 4 °C) to remove cellular debris and ultracentrifugation at 105,000 g for 1 h at 4 °C, the cytosoluble proteins were stored at −80 °C until analysis, and total protein concentration in each sample was determined using the Bradford Protein Assay Kit (Biorad, Marnes-la-Coquette, France) according to the manufacturer’s instructions.

#### Phosphorylated peptides enrichment

For each biological replicate, 4 mg of proteins were reduced with 13.3 mM DTT for 30 min at 37 °C and alkylated with 42 mM iodoacetamide for 30 min at room temperature, before digestion with 40 µg of trypsin (modified, sequencing grade, Promega, Charbonnières, France) overnight at 37 °C. Tryptic peptides were then desalted using Sep-Pak tC18 columns (Waters, Saint-Quentin, France) according to the manufacturer’s instructions. Phosphorylated peptides were enriched with SIMAC (Sequential elution from IMAC) adapted from Thingholm *et al*. method^18^, using a combination of the Pierce® Fe-NTA Phopshopeptide Enrichment kit (ThermoFisherScientific) and the Pierce® TiO_2_ Phosphopeptide Enrichment kit according to the manufacturer’s instructions with some modifications. Briefly tryptic peptides were loaded onto the Fe-NTA spin column and three fractions were recovered: (1) the first fraction was eluted with acid conditions (1% trifluoroacetic acid/20% acetonitrile), (2) the second fraction was eluted with basic conditions (Elution Buffer from the Fe-NTA kit), and (3) the unbound flow through fraction was further processed by the TiO_2_ Spin Tip. After each of these three steps, phosphorylated peptide fractions were concentrated and desalted again using the Pierce® Graphite Spin Columns according to the manufacturer’s instructions. The experimental approach is outlined in Fig. 1.

#### Mass spectrometry analysis

Mass spectrometry analysis of each eluted fraction was performed using a nanoflow high-performance liquid chromatography (HPLC) system (Dionex, LC Packings Ultimate 3000) connected to a hybrid LTQ-OrbiTrap XL (Thermo Fisher Scientific) equipped with a nano-electrospray ionization (ESI) source (New Objective). The LTQ-Orbitrap XL instrument was operated in the data-dependent mode by automatically switching between full scan MS and consecutive MS/MS acquisitions. Three different methods were used for each elution fraction: a classical Collision-Induced Dissociation (CID), a Neutral Loss (NL) and a Multi-Stage Activation (MSA). For each method, full scan MS spectra were acquired in the OrbiTrap with a resolution of 60000 at m/z 400 in the mass range 400–2000; ion injection times were calculated to allow the accumulation of 5.10^5^ ions in the OrbiTrap for each spectrum. The ten most intense ions (with an intensity ≥ 2000 counts for CID and NL methods or 5000 for MSA method and a charge state ≥ 2) of each full scan MS were sequentially isolated (precursor selection window: 2 Da) and fragmented in the linear ion trap by collision-induced dissociation. For the NL method, masses specified for further CID fragmentation were 32.6, 49, 65.3, 98 and 147 Da with a precursor selection window of 2 Da. For the MSA method, neutral loss masses specified for further MSA fragmentation of the precursor were 24.49, 26.65, 32.65, 39.98 and 48.98 Da with a precursor selection window of 3 Da. For OrbiTrap measurements, an external calibration was used before each injection series ensuring an overall error mass accuracy below 5 ppm for the detected ions. MS data were saved in RAW file format (Thermo Fisher Scientific) using XCalibur 2.0.7 with tune 2.4. The experimental approach is outlined in Fig. 1.

#### Protein identification

Proteome Discoverer 1.2 software (Thermo Fisher Scientific) supported by Mascot (Mascot server v2.2.07; http://www.matrixscience.com) database search engine was used for peptide and protein identification using its automatic decoy database search to calculate a false discovery rate (FDR). MS/MS spectra were compared to the UniProt *D. melanogaster* reference proteome database (UniProt UP000000803 release May 14, 2016, 22023 sequences). Mass tolerance for MS and MS/MS was set at 10 ppm and 0.5 Da, respectively. The enzyme selectivity was set to full trypsin with one miscleavage allowed. Protein modifications were fixed carbamidomethylation of cysteines, variable oxidation of methionine, variable acetylation of N-terminus and variable phosphorylation of serine, threonine or tyrosine.

#### Identification validation and spectral count label-free quantification

Proline Studio 1.3 software was used for the validation and the spectral count comparisons of the identified proteins in each samples (http://proline.profiproteomics.fr/)^53^. After importation of the mascot. dat files from each query, each search results were validated with a peptide rank = 1 and a FDR of 1% on mascot score at the peptide spectrum match (PSM) level. All the identification summaries (*i.e*., the validated Mascot search results) of all the MS analyses of all the elution fractions corresponding to one biological replicate were merged and a protein inference list was created. Proteins identified with exactly the same set of peptides or with a subset of the same peptides were grouped in a protein set. This protein set was then represented by a typical protein, which is the best identified protein (best score) or in case of same set proteins, the SwissProt one if possible. For the spectral count comparison, a parent dataset corresponding to the merge of the individual biological replicate identification summaries was created to define the shared and specific peptides and the protein set list to compare (CACO protein set, Table S1). For each protein, weighted spectral counts were calculated, as suggested in Abacus^54^, where shared peptides are combined and weighted according to the associated protein sets. To detect significant difference between the two conditions (CO vs. CA) a beta-binomial test was performed on the weighed spectral counts calculated for each biological sample and a *p*-value was calculated for each protein set using the R package BetaBinomial 1.2 implemented in Proline Studio^55^. For each protein, an expression ratio was calculated between CA and CO samples, which represented the ratio of the mean of the weighted spectral counts in the four CA replicates to the mean of the weighted spectral counts in the four CO replicates. In order to be able to calculate such ratio, even when a protein is not identified in one condition, one spectral count is added to the whole weighted spectral counts, meaning that when a protein is not identified in one sample its weighted spectral count is equal to one.

#### Data availability

The raw mass spectrometry proteomics data have been deposited with the ProteomeXchange Consortium^56^ *via* the PRIDE partner repository^57^ with the dataset identifier PXD005311 and doi:10.6019/PXD005311.

#### Datamining

We used InteractiVenn tool to detect and visualize the amount of overlap between lists of proteins from both treatmetnts^58^. From this, were retrieved a set of phosphoproteins that were unique in CA, another set where phosphoproteins were lacking in CA (only found in CO), and a common set of phosphoproteins that were detected in both treatments. Non-phosphorylated proteins were not considered in further analyses. To reveal possible protein-protein interactions, sets of phosphoproteins of interest [list_CA^(+)^ and list_CA^(−)^] were used to query STRING database^23^. The STRING algorithm links proteins into networks based on published functional or informatics-predicted interactions^23^. Default parameters were used except that we increased the minimum required interaction score to 0.7 (high confidence). Also, to increase visibility, disconnected nodes in the network were not displayed. Gene Ontology (GO) enrichment analyses were performed in STRING (considering FDR < 0.05). Non-redundant and significant overrepresented GO-terms were then reduced and visualized using REVIGO program with the default similarity value (0.7) and Resnik (normalized) as semantic similarity measure^24^. Data were then exported in R statistical software (v.3.0.3.) to generate treemap views using the “treemap” package, in which related terms were joined into loosely related “superclusters” visualized with different colors. In treemaps, the size of the rectangles was adjusted to reflect the *p*-values of the GO-terms.

## Electronic supplementary material

Supplementary dataset Table S-1

Supplementary dataset Table S-2

Supplementary dataset Table S-3

Supplementary dataset Table S-4

Supplementary dataset Table S-5
